# Successful weight reduction and maintenance by using a smartphone application in those with overweight and obesity

**DOI:** 10.1038/srep34563

**Published:** 2016-11-07

**Authors:** Sang Ouk Chin, Changwon Keum, Junghoon Woo, Jehwan Park, Hyung Jin Choi, Jeong-taek Woo, Sang Youl Rhee

**Affiliations:** 1 Department of Internal Medicine, Jeju National University School of Medicine, Jeju, Korea; 2 Division Biomedical Research Institute, Geference Inc., Seoul, Korea; 3 Data and Analytics, KPMG LLP, New York, New York, USA; 4 Department of Anatomy, Seoul National University College of Medicine, Seoul, Korea; 5 Department of Endocrinology and Metabolism, Kyung Hee University School of Medicine, Seoul, Korea

## Abstract

A discrepancy exists with regard to the effect of smartphone applications (apps) on weight reduction due to the several limitations of previous studies. This is a retrospective cohort study, aimed to investigate the effectiveness of a smartphone app on weight reduction in obese or overweight individuals, based on the complete enumeration study that utilized the clinical and logging data entered by Noom Coach app users between October 2012 and April 2014. A total of 35,921 participants were included in the analysis, of whom 77.9% reported a decrease in body weight while they were using the app (median 267 days; interquartile range = 182). Dinner input frequency was the most important factor for successful weight loss (OR = 10.69; 95% CI = 6.20–19.53; p < 0.001), and more frequent input of weight significantly decreased the possibility of experiencing the yo-yo effect (OR = 0.59, 95% CI = 0.39–0.89; p < 0.001). This study demonstrated the clinical utility of an app for successful weight reduction in the majority of the app users; the effects were more significant for individuals who monitored their weight and diet more frequently.

Obesity is a global epidemic with a rapidly increasing prevalence worldwide^1^,^2^. As obese individuals experience significantly higher mortality when compared with the non-obese population^3^,^4^, this phenomenon poses a significant socioeconomic burden, necessitating strategies to manage overweight and prevent obesity^5^. Although numerous interventions such as life style modification including exercise^6^,^7^,^8^,^9^,^10^, and pharmacotherapy^11^,^12^,^13^ have been shown effective for both the prevention and treatment of obesity, some of these methods were found to have a limitation which required substantial financial inputs and repeated time-consuming processes^14^,^15^.

Recently, as the number of smartphone users is increasing dramatically, many investigators have attempted to implement smartphone applications (app) for health promotion^16^,^17^,^18^,^19^. Consequently, many smartphone apps have demonstrated at least partial efficacy in promoting successful weight reduction according to the number of previous studies^20^,^21^,^22^,^23^,^24^. However, due to the limitations associated with study design such as small-scale studies and short investigation periods, a discrepancy exists with regard to the effect of apps on weight reduction^20^,^21^,^23^. Even systemic reviews which investigated the efficacy of mobile apps for weight reduction reported more or less inconsistent results; Flores Mateo *et al*. reported a significant weight loss by mobile phone app intervention when compared with control groups^25^ whereas Semper *et al*. reported that four of the six studies included in the analysis showed no significant difference of weight reduction between comparison groups^26^. Thus, the aim of this study was to investigate the effectiveness of a smartphone app on weight reduction in obese or overweight individuals using data collected from one of the most widely used weight loss apps.

## Methods

### Noom Coach

Noom Coach (Noom Inc., New York, NY, USA) is one of the most popular publicly available apps for weight loss. This app has been the top grossing health and fitness app in the Google Play store since 2012 with more than 10 million installs worldwide, and it has been consistently ranked as one of the top weight loss apps^27^,^28^. At the first log-on, users are asked to set their target body weight and to record their current body weight. As using the app, the users record their daily food intake, and an activity monitor embedded in the app daily saves the number of steps taken per day. Based on these data, the app generates reports demonstrating the user’s weight trends, as well as calorie and nutritional summaries of their diet, and provides feedback, including the types of exercises that help achieve the user’s target body weight.

### Study protocol

This is a retrospective cohort study designed to investigate the effect of the Noom Coach smartphone app on weight reduction and to identify independent factors influencing long-term success or failure and maintenance of weight reduction after using the smartphone app. The analyses in this study were based on a complete enumeration of Noom Coach app users who installed the app between October 10, 2012 and April 9, 2014. The investigators were provided with all relevant data by the app company following de-identification of the entire dataset. The app company had no role in the development of the protocol, the interpretation of the data, or the preparation of the manuscript. All app users who logged in and recorded their data two or more times a month for 6 consecutive months were included in the study. For the purpose of accurate analysis, we excluded users exhibiting the top 1% of weight variances and the top and bottom 0.5% of other variables, along with user-defined inputs deemed to be unrealistic outliers. Users who were at the age of 42 years were also excluded, as the default age of this app is 42 years, and many users chose not to change the default to their actual age when using the app.

We analyzed data including gender, age, height, weight, diet-related variables (input frequency for breakfast, lunch and dinner, and calories for breakfast, lunch and dinner), exercise-related variables (exercise input frequency and related calorie expenditure) and variables regarding weight change (weight input frequency and final body mass index, BMI). Input frequency represented how often the app user participated in self-monitoring, defined as the number of reports recorded per day in the app about body weight daily measured as well as meals and exercise which the participant daily ingested and performed. Changes of body weight was defined as its change while the participants were using the app in comparison with their baseline body weight.

To investigate the long-term efficacy of the app, as well as factors influencing the maintenance of weight loss, the study period was divided to two phases: initial (0–26 weeks) and long-term (27 to study end [76 weeks]). Also, app users included in the analyses were classified according to their weight loss patterns: success, partial success, stationary and yo-yo groups (for the definitions of each group, see Appendix Table 1). In addition, users with >20% missing data were further excluded from analyses associated with weight loss patterns.

### Statistical Analyses

We assessed the effectiveness of weight loss using the paired *t*-test between the baseline and final weights of participants. To compare the user’s weight loss regardless of duration of participation, we normalized the weight loss by the number of participation days and examined the association between weight loss and the independent variables using both univariate and multivariate analyses. For the variables with a significance level of *p* < 0.2 in the univariate analysis, multivariate analyses were performed to investigate their independent association with the likelihood of weight loss. The direction and significance of association were examined using the regression coefficient β and the Wald test for the regression coefficient. Simple linear regression was used to examine the statistical significance of a linear relationship between the observation time and weight loss in the four groups, success, partial success, yo-yo and stationary. Factors contributing to the likelihood of experiencing successful weight loss (either in the success or partial success group) or the yo-yo effect (in the yo-yo group) were examined using simple and multiple logistic regression models. The stationary group was used as a reference. Variables which showed moderate statistical significance (p < 0.2) in the univariate analysis were examined for their independent contribution using multivariate logistic regression. All statistical analyses were conducted using R version 2.14.2^29^.

### Ethics Statement

This study was conducted in accordance with the guidelines laid down in the Declaration of Helsinki, the privacy policy of Noom Inc., and approved by the Kyung Hee University Hospital Institutional Review Board (KMC IRB 1435-04), which confirmed the absence of risk for the de-identified personal information leakage. The informed consents from the subjects were waived by the KMC IRB due to the retrospective design of this study.

## Results

### Effectiveness of the smartphone app on weight loss

**Figure 1:**
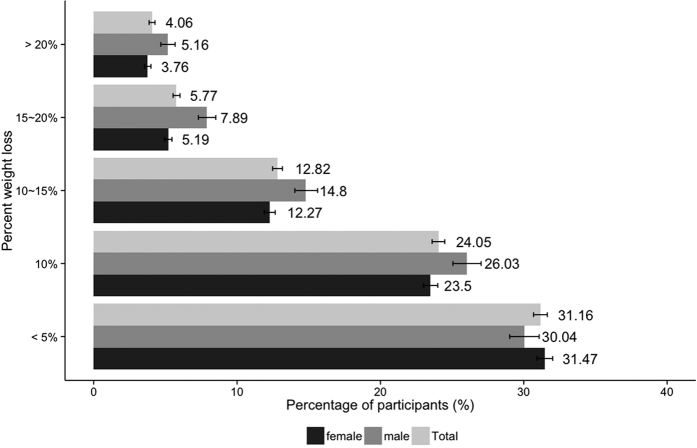
Distribution of weight loss among app users. Percentages (and 95% CIs) of participants achieving <5%, 5–10%, 10–15%, 15–20% and >20% weight loss relative to baseline at the end of the 6-month trial period. Data are reported as the mean ± SD.

Baseline characteristics of all participants are shown in Table 1, and the global distribution of the participants is shown in Appendix Table 2. A total of 35,921 participants (M:F = 21.6%: 78.4%) were included in the analysis. The median duration of app usage was 267 (interquartile range (IQR) = 182) fdays, with male participants using the app longer compared with females (276 *vs*. 264 days, Table 2). Baseline BMI was 30.2 ± 0.1 kg/m^2^ for males and 28.0 ± 0.0 kg/m^2^ for females. Among the participants, 77.9% reported a decrease in body weight while they were using the app with 22.7% experiencing more than 10% weight loss compared with baseline with a higher weight loss success rate in males (83.9 *vs*. 76.1%, Fig. 1), resulting in final BMIs of 28.1 ± 0.1 kg/m^2^ for males and 26.5 ± 0.0 kg/m^2^ for females (Table 2).

### Factors contributing to weight loss

The individual variables contributing to weight loss were assessed using univariate linear regression. Variables found to contribute to weight loss were then analyzed by multivariate linear regression. Gender, baseline BMI, weight input frequency (*β* = 2.01, 95% confidence interval [CI] = 1.85–2.17, p < 0.001), exercise, and dinner input frequency (*β* = 2.56, 95% CI = 2.27–2.85, p < 0.001, Table 3) were all positively and significantly correlated with the amount of weight reduction, while age, breakfast input frequency, breakfast calories, lunch calories and dinner calories exhibited significant negative correlations (Table 3).

### Differential weight change patterns

To analyze according to the app user’s weight change patterns, data from 15,376 participants (M:F = 3,761:11,615) were included after further excluding those with >20% missing data (Appendix Table 2). Those classified either in the success or partial success group experienced an obvious significant linear weight reduction (Appendix Table 3), which was maintained throughout the remainder of the study period, while participants either in the stationary or yo-yo group did not show a significant weight change at the end of the study period. The weight loss in the yo-yo group was comparable with that in the partial success group during the critical period (7.557 ± 0.387 and 5.899 ± 0.252 kg for males and females, respectively), although the weight loss was regained or even exceeded the baseline weight at the end of the study period (Appendix Fig. 1). Interestingly, the rate of weight loss was faster in the yo-yo group than in the partial success group during the first 8 weeks (Appendix Table 4).

### Factors contributing to weight loss in the Successful and Yo-yo groups

The results of the analysis regarding the factors which contributed to successful weight loss are shown in Table 4. Gender (male), younger age, higher baseline BMI, weight input frequency, breakfast and dinner input frequencies, breakfast, lunch and dinner calories, exercise input frequency and related calorie expenditure remained significant in the multivariate analysis. Breakfast input frequency was significant in the multivariate analysis, but the direction was reversed. Dinner input frequency was the most important factor for successful weight loss (odds ratio [OR] = 10.69; 95% CI = 6.20–19.53; p < 0.001).

Regarding the variables affecting the yo-yo effect, gender (male), younger age and baseline BMI remained significant in the multivariate analysis (Appendix Table 5). In addition, more frequent input of weight significantly decreased the possibility of experiencing the yo-yo effect (OR = 0.59, 95% CI = 0.39–0.89; p < 0.001). Calorie intake from lunch and dinner also showed statistically significant associations in the multivariate analysis.

## Discussion

In the present study we utilized the data from 35,921 Noom app users and found that 77.9% of study participants reported a decrease in body weight while they were using the app (median duration of app usage = 267 (IQR = 182) days). BMI changed from 30.2 ± 0.1 to 28.1 ± 0.1 kg/m^2^ for males and 28.0 ± 0.0 to 26.5 ± 0.0 kg/m^2^ for females, with 22.7% of all app users experiencing >10% weight reduction compared with baseline. Weight reduction due to app use was expected to be greater in males and in those with high baseline BMI and more frequent inputs for weight, exercise and dinner. The most important factor affecting maintenance or failure of weight reduction was the dinner input frequency.

As the prevalence of overweight and obesity increases and its socioeconomic costs escalate dramatically, various pharmacological and surgical interventions have been developed to manage and prevent obesity. The Diabetes Prevention Program (DPP)-intensive lifestyle intervention is one such method, designed to produce clinically significant weight reduction in adults with prediabetes, proving its effectiveness for both weight loss and cardiometabolic outcomes^8^. In addition, life style modification has been shown to be effective for reducing body weight and cardiovascular risk^6^,^7^,^8^,^9^,^10^; however, each of these studies had important limitations, particularly in that some of them were resource intensive, expensive, and time-consuming^14^,^15^. Frequent group and individual in-person counselling and communication were also required for successful outcomes, representing significant barriers to more active participation^21^.

Lifestyle modification through the use of cognitive and behavioral treatments has long been regarded as one of the most effective tools for maintaining weight loss in overweight and obese individuals^30^. Among the various behavioral strategies, weight self-monitoring has proven effective for weight reduction and maintenance^31^,^32^. However, because the traditional method of self-monitoring relies on the use of a paper diary, the rather tedious and time-consuming nature of this approach, combined with a time-lag for motivational feedback, limits its overall effectiveness^33^,^34^. Recently, the field of mobile apps is growing rapidly, with an estimated 10,000 globally available apps targeting diet and weight loss^35^. Due to their ubiquity, these apps enable better adherence than do paper diaries^20^, making smartphone apps an appealing alternative for cognitive and behavioral treatment on obesity and overweight, which would be able to overcome some of the limitations of classical weight-loss programs. Despite lower overall energy intake and more physical activity than those of non-users^36^,^37^, there are still inconsistent findings with respect to the effect of apps on weight reduction^20^,^21^,^23^,^25^,^26^.

On the other hand, in the present study, we demonstrated significant weight reduction in a majority of app users, and found out that the most critical factor in determining either successful weight reduction or the yo-yo effect was the input frequencies of diet, weight, and exercise. This finding highlights the importance of recording and managing factors associated with one’s daily lifestyle, which is consistent with the well-known classical concept that regular and frequent self-monitoring of weight, physical activity and calories from diet is a key factor leading to successful weight loss^34^,^38^. In addition, the present study specifically demonstrated which aspects of daily life need to be recorded and monitored frequently to achieve effective body weight reduction. Of these factors, the most important criterion was dinner input frequency, which provides additional evidence for the significance of dinner in weight gain and obesity^39^,^40^. Unexpectedly, dietary calorie intake did not play a significant role in weight reduction, possibly because the food database embedded in apps may not accurately reflect the food calories entered by app users worldwide. Previous reports showed that many apps for weight reduction did not fully incorporate evidence-based behavioral strategies^27^, possibly causing inaccurate calculation of food calories entered by app users. Also, the follow-up days was found not to have a significant effect on the successful weight loss and maintenance. It has been reported that the initial weight loss during the early period of treatment either with life style modification or pharmacologic intervention for obesity and overweight would be critical for maintaining the weight loss^41^,^42^, which is consistent with our findings despite the lack of previous studies which investigated the association between apps for weight loss and initial response.

Another interesting finding was that weight reduction was greater in male users, primarily due to the higher baseline BMI and therefore potentially greater motivation among male users. However, it should be addressed that gender difference remained significant after multivariate analysis, which implies the possibility of different app use patterns between genders; male users in the present study showed a higher frequency of data input than that of female users, which is in agreement with previous reports^36^. The greater adherence of male users to the app is believed to contribute to more effective weight loss (Table 3). A larger scaled study is necessary to re-evaluate our findings. The younger users were also found to benefit more from using an app, because mobile devices are more popular in this segment of the population^43^. Together with the significance of dinner input frequency, these findings clearly highlight the importance of adherence to an app for successful weight reduction.

Among the most noteworthy effects observed in this study was the large gender disparity associated with usage patterns. The majority of male users at baseline were categorized as either overweight or obese class I, whereas female users were mostly normal or overweight (Table 1). A similar gender disparity was previously reported by Rhee and colleagues, who showed that the prevalence of obesity in Korea increases continuously with age in males but decreases in young and middle-aged females^44^. Consistent with this finding, our result globally represent how males and females recognize and react differently to their body weight status.

The present study had several limitations associated with this study. First, this was not a randomized, controlled trial (RCT) and thus comparisons with a control group could not be made. However, most of the previous studies on apps were usually limited by high attrition rates; those assigned to an app user group were not likely to continue to use the app if their weight reduction was not satisfactory during the early study period^20^,^21^,^23^. Contamination of the control group was also common in previous studies, as participants in the control group rather used the study app during the trial^21^,^23^, which may affect the reliability of these RCTs. To solve this limitation, the present study utilized data entered only by the participants who satisfied the inclusion criteria, though it is not certain what percentage of the entire app users were actually included in this study. There must be further studies which would be able to mitigate potential sources of such bias which had been found in previous and present studies. Second, all the data including food and exercise were self-reported and prone to social desirability which may lead to inaccurate data. The database of food calories embedded in the app also may not be as accurate as expected. This is one of the limitations reported previously^27^. However, it should be addressed that our study demonstrated the importance of adherence to the app represented by the frequency of using the app for the successful weight loss even without the accurate calculation of food and exercise calories. Lastly, there exists some variability of the utility of BMI in different regions of the world, addressing some limitation of BMI as a global standard to evaluate the efficacy of treatment for obesity or overweight; it has been reported that Asians have a higher percentage of abdominal fat and intramyocellular lipid and liver fat content when compared with Caucasians^45^. Different ethnic groups in Asia such as Asian Indians, Malay and Chinese having the same BMI may show different body fat ratio^45^. However, to the best of our knowledge, this study is the first cohort study to demonstrate the effect of an app on weight reduction, to define the characteristics of a user group most likely to benefit from the app, and to identify specific aspects of daily life (input frequency for dinner) that should be intensively monitored for achievement and maintenance of weight reduction. Based on these findings, developing methods to encourage the use of apps to achieve goals would add functionality of mobile technology leading to more effective weight reduction and obesity prevention in the future.

In conclusion, we demonstrated clinical utility of a smartphone app for successful weight reduction in the majority of the app users, which was more significant for individuals who monitored their weight and diet more frequently. Further studies will be necessary to develop methods for encouraging adherence to self-monitoring apps.

## Additional Information

**How to cite this article**: Chin, S. O. *et al*. Successful weight reduction and maintenance by using a smartphone application in those with overweight and obesity. *Sci. Rep.* **6**, 34563; doi: 10.1038/srep34563 (2016).

**Publisher’s note**: Springer Nature remains neutral with regard to jurisdictional claims in published maps and institutional affiliations.

## Supplementary Material

Supplementary Information

## Figures and Tables

**Table 1:** Baseline Characteristics of the Study Participants.

	Male(n = 7,734)	Female(n = 28,097)	Total(n = 35,831)
Age (years)	37.6 ± 0.1	32.2 ± 0.1	33.3 ± 0.1
Height (cm)	177.5 ± 0.1	164.7 ± 0.0	167.4 ± 0.1
Weight (kg)	95.3 ± 0.2	76.2 ± 0.1	80.3 ± 0.1
Baseline BMI (kg/m^2^)*	30.2 ± 0.1	28.0 ± 0.0	28.5 ± 0.0
Underweight (BMI < 18.5)	0.1%	1.0%	0.8%
Normal (18.5~25)	11.4%	38.4%	32.6%
Overweight (25~30)	45.5%	30.0%	33.4%
Obesity class I (30~35)	28.2%	16.5%	19.0%
Obesity class II (35~40)	9.8%	7.9%	8.3%
Obesity class III (>40)	5.0%	6.2%	6.0%

Data are reported as the mean ± SD, unless otherwise stated. All variables showed statistically significant difference between male and female (p < 0.001).

^*^BMI classification is based on the WHO criteria^46^. Abbreviations: BMI, body mass index.

**Table 2:** Clinical Course of the Study Participants.

	Male (n = 7,734)	Female (n = 28,097)	Total (n = 35,831)
Follow-up days	Median = 276 (IQR = 196.75)	Median = 264 (IQR = 178)	Median = 267 (IQR = 182)
Person-day	2,499,628	8,587,366	11,086,994
Diet related variables			
Breakfast input frequency (n/person-day)	0.455 ± 0.004	0.435 ± 0.002	0.439 ± 0.002
Lunch input frequency (n/person-day)	0.418 ± 0.003	0.4 ± 0.002	0.404 ± 0.002
Dinner input frequency (n/person-day)	0.355 ± 0.003	0.333 ± 0.002	0.338 ± 0.001
Breakfast calories (kcal/person-day)	325.5 ± 1.4	275.8 ± 0.6	286.5 ± 0.6
Lunch calories (kcal/person-day)	491.0 ± 1.7	393.1 ± 0.7	414.3 ± 0.7
Dinner calories (kcal/person-day)	567.4 ± 2.1	432.5 ± 0.8	461.6 ± 0.8
Exercise related variables			
Exercise input frequency (n/person-day)	0.244 ± 0.002	0.225 ± 0.001	0.229 ± 0.001
Exercise calories expenditure (kcal/person-day)	407.907 ± 2.573	277.442 ± 0.937	305.602 ± 0.964
Change of weight			
Weight input frequency (n/person-day)	0.27 ± 0.002	0.244 ± 0.001	0.25 ± 0.001
Final BMI (kg/m^2^)	28.1 ± 0.1	26.5 ± 0.0	26.8 ± 0.0
Underweight (<18.5)	0.2%	2.2%	1.7%
Normal (18.5~25)	27.1%	48.2%	43.7%
Overweight (25~30)	45.7%	26.9%	31.0%
Obesity class I (30~35)	18.6%	12.8%	14.1%
Obesity class II (35~40)	5.7%	6.0%	5.9%
Obesity class III (>40)	2.6%	3.9%	3.6%

Data are reported as the mean ± SD, unless otherwise stated. All variables showed statistically significant difference between male and female (p < 0.001).

Abbreviations: BMI, body mass index; IOR, interquartile range.

**Table 3:** Factors contributing to the weight loss.

	Univariate Linear Regression	p-value	Multivariate Linear Regression	p-value
	***β*** (95% CI)		***β*** (95% CI)	
Gender (male)	0.60 (0.54, 0.66)	<0.001	0.71 (0.65, 0.77)	<0.001
Age	0.01 (0.008, 0.013)	<0.001	−0.026 (−0.03, −0.02)	<0.001
Follow-up Days	−0.001 (−0.001, −0.001)	<0.001	0.00 (0.00, 0.00)	0.886
Baseline BMI	0.146 (0.143, 0.150)	<0.001	0.165 (0.161, 0.168)	<0.001
Weight input frequency	2.45 (2.30, 2.61)	<0.001	2.01 (1.85, 2.17)	<0.001
Breakfast input frequency	1.39 (1.31, 1.48)	<0.001	−1.02 (−1.27, −0.78)	<0.001
Lunch input frequency	1.58 (1.49, 1.67)	<0.001	−0.15 (−0.52, 0.21)	0.418
Dinner input frequency	1.84 (1.74, 1.93)	<0.001	2.56 (2.27, 2.85)	<0.001
Breakfast calories	−0.001 (−0.001, −0.001)	<0.001	−0.001 (−0.002, −0.001)	<0.001
Lunch calories	−0.001 (−0.001, −0.001)	<0.001	−0.001 (−0.001, −0.001)	<0.001
Dinner calories	0.00 (0.00, 0.00)	0.0358	−0.002 (−0.002, −0.001)	<0.001
Exercise input frequency	1.75 (1.63, 1.88)	<0.001	0.72 (0.59, 0.85)	<0.001
Exercise calories expenditure	0.001 (0.001, 0.001)	<0.001	0.00 (0.00, 0.00)	<0.001

Abbreviations: BMI, body mass index; OR, odds ratio; CI, confidence interval.

**Table 4:** Factors contributing to being a success or a partial success against stationary subgroup.

	Univariate Logistic Regression	Wald Test p-value	Multivariate Logistic Regression	Wald Test p-value
	OR (95% CI)		OR (95% CI)	
Gender (male)	1.44 (1.29, 1.60)	<0.001	2.05 (1.79, 2.36)	<0.001
Age	0.99 (0.99, 1.00)	0.002	0.97 (0.95, 0.97)	<0.001
Follow-up Days	1.00 (1.000, 1.00)	0.627	—	—
Baseline BMI	1.10 (1.09, 1.11)	<0.001	1.13 (1.12, 1.14)	<0.001
Weight input frequency (n/person-day)	2.85 (2.20, 3.70)	<0.001	3.0 (2.21, 4.08)	<0.001
Breakfast input frequency (n/person-day)	3.15 (2.72, 3.66)	<0.001	0.36 (0.22, 0.57)	<0.001
Lunch input frequency (n/person-day)	3.98 (3.42, 4.64)	<0.001	1.14 (0.57, 2.28)	0.718
Dinner input frequency (n/person-day)	4.86 (4.16, 5.68)	<0.001	10.69 (6.20, 18.53)	<0.001
Breakfast calories (kcal/person-day)	1.00 (1.00, 1.00)	<0.001	1.00 (1.00, 1.00)	<0.001
Lunch calories (kcal/person-day)	1.00 (1.00, 1.00)	<0.001	1.00 (1.00, 1.00)	<0.001
Dinner calories (kcal/person-day)	1.00 (1.00, 1.00)	0.105	1.00 (1.00, 1.00)	<0.001
Exercise input frequency (n/person-day)	4.02 (3.30, 4.90)	<0.001	2.49 (1.96, 3.17)	<0.001
Exercise calories expenditure (kcal/person-day)	1.00 (1.00, 1.00)	<0.001	1.00 (1.00, 1.00)	0.085

Abbreviations: BMI, body mass index; OR, odds ratio; CI, confidence interval.
