# Supplementary material for: Successful weight reduction and maintenance by using a smartphone application in those with overweight and obesity
Source: Sci Rep. 2016 Nov 7;6:34563. doi: 10.1038/srep34563 (PMC5098151; doi:10.1038/srep34563)
Supplement: Supplementary Information [file srep34563-s1.doc]

**Title:** Successful weight reduction and maintenance by using a smartphone application in those with overweight and obesity

**Author:** Sang Ouk Chin, M.D., Ph.D.^1¶^, Changwon Keum, M.S.^2¶^, Junghoon Woo, Ph.D.^3^, Jehwan Park, Ph.D.^2^, Hyung Jin Choi, M.D., Ph.D.^4^, Jeong-taek Woo, M.D., Ph.D.^5^, Sang Youl Rhee, M.D., Ph.D.^5^

**Affiliations:**

^1^Department of Internal Medicine, Jeju National University School of Medicine, Jeju, Korea

^2^Division Biomedical Research Institute, Geference Inc., Seoul, Korea

^3^Data and Analytics, KPMG LLP, New York, New York, USA

^4^Department of Anatomy, Seoul National University College of Medicine, Seoul, Korea

^5^Department of Endocrinology and Metabolism, Kyung Hee University School of Medicine, Seoul, Korea

**Corresponding Author:** Sang Youl Rhee, M.D., Ph.D.

Department of Endocrinology and Metabolism, Kyung Hee University School of Medicine,

#26 Kyungheedae-ro, Dongdaemoon-gu, Seoul, Korea (02447)

Phone: +82-2-958-8200

Fax Number: 82-2-968-1848

E-mail: bard95@hanmail.net

**Abbreviated Title:** smartphone application and weight loss

**Keywords:** weight loss, smartphone, application

^¶^The first two authors contributed equally to this work.

**Table 1.** Baseline characteristics in success, partial success, stationary, and yo-yo subgroups

|  | success | | | | partial success | | | | stationary | | | | yo-yo | | | |
| --- | --- | --- | --- | --- | --- | --- | --- | --- | --- | --- | --- | --- | --- | --- | --- | --- |
|  | Male (n=615) | Female (n=1543) | P | Total (N=2158) | Male (n=327) | Female (n=1134) | P | Total (N=1461) | Male (n=805) | Female (n=3300) | P | Total (N=4105) | Male (n=389) | Female (n=1334) | P | Total (N=1723) |
| 1) At baseline |  |  |  |  |  |  |  |  |  |  |  |  |  |  |  |  |
| Age | 38.5±0.9 | 34.5±0.5 | <0.001 | 35.6±0.5 | 39.5±1.2 | 34.8±0.7 | <0.001 | 35.8±0.6 | 41.8±0.8 | 35.2±0.4 | <0.001 | 36.5±0.4 | 39.8±1.0 | 33.3±0.6 | <0.001 | 34.7±0.5 |
| Height | 178.1±0.6 | 165.4±0.3 | <0.001 | 169.0±0.4 | 177.8±0.8 | 164.6±0.4 | <0.001 | 167.5±0.5 | 177.0±0.5 | 164.6±0.2 | <0.001 | 167.0±0.3 | 177.1±0.7 | 164.8±0.4 | <0.001 | 167.6±0.4 |
| Weight | 106.2±1.7 | 88.0±1.0 | <0.001 | 93.1±1.0 | 96.4±2.1 | 79.4±1.1 | <0.001 | 83.2±1.1 | 92.0±1.3 | 72.7±0.7 | <0.001 | 76.4±0.6 | 93.3±1.7 | 74.0±1.0 | <0.001 | 78.4±0.9 |
| Baseline BMI | 33.4±0.5 | 32.1±0.4 | <0.001 | 32.5±0.3 | 30.4±0.6 | 29.3±0.4 | 0.002 | 29.5±0.3 | 29.2±0.4 | 26.7±0.2 | <0.001 | 27.2±0.2 | 29.7±0.5 | 27.2±0.3 | <0.001 | 27.8±0.3 |
| Underweight  (< 18.5) | 0 | 0.001 | - | 0 | 0 | 0.004 | - | 0.003 | 0.002 | 0.02 | <0.001 | 0.017 | 0.003 | 0.012 | 0.001 | 0.01 |
| Normal (18.5~25) | 0.037 | 0.135 | <0.001 | 0.108 | 0.092 | 0.272 | <0.001 | 0.231 | 0.188 | 0.473 | <0.001 | 0.417 | 0.136 | 0.44 | <0.001 | 0.371 |
| Overweight (25~30) | 0.265 | 0.312 | <0.001 | 0.299 | 0.489 | 0.348 | <0.001 | 0.38 | 0.455 | 0.263 | <0.001 | 0.3 | 0.463 | 0.282 | 0.439 | 0.323 |
| Obesity class I (30~35) | 0.376 | 0.269 | 0.024 | 0.299 | 0.269 | 0.206 | <0.001 | 0.22 | 0.232 | 0.133 | 0.002 | 0.152 | 0.254 | 0.151 | 0.888 | 0.174 |
| Obesity class II (35~40) | 0.193 | 0.149 | 0.644 | 0.162 | 0.098 | 0.093 | <0.001 | 0.094 | 0.084 | 0.065 | <0.001 | 0.068 | 0.095 | 0.07 | 0.062 | 0.075 |
| Obesity class III (>40) | 0.128 | 0.134 | 0.001 | 0.132 | 0.052 | 0.078 | <0.001 | 0.072 | 0.039 | 0.046 | <0.001 | 0.045 | 0.049 | 0.046 | 0.005 | 0.046 |
| 2) During follow-up |  |  |  |  |  |  |  |  |  |  |  |  |  |  |  |  |
| Follow-up days | 290 (IQR = 154) | 279 (IQR = 151.5) | 0.128 | 330.537±4.964 | 311 (IQR = 160) | 295 (IQR = 167.75) | 0.242 | 344.807±6.526 | 314 (IQR = 158) | 291 (IQR = 154) | 0.004 | 334.951±3.705 | 413 (IQR = 246) | 379 (IQR = 230) | <0.001 | 405.802±6.498 |
| Person-day | 207,092 | 506,207 | - | 713,299 | 115,189 | 388,574 | - | 503,763 | 278,538 | 1,096,434 | - | 1,374,972 | 167,355 | 531,842 | - | 699,197 |
| * diet related variables |  |  |  |  |  |  |  |  |  |  |  |  |  |  |  |  |
| Weight input frequency | 0.376±0.016 | 0.351±0.009 | 0.008 | 0.358±0.008 | 0.339±0.019 | 0.321±0.009 | 0.09 | 0.325±0.008 | 0.331±0.013 | 0.309±0.005 | 0.002 | 0.313±0.005 | 0.273±0.015 | 0.274±0.008 | 0.864 | 0.274±0.007 |
| Breakfast input frequency (n/person-day) | 0.643±0.024 | 0.635±0.015 | 0.589 | 0.638±0.013 | 0.59±0.034 | 0.573±0.018 | 0.403 | 0.577±0.016 | 0.495±0.021 | 0.508±0.01 | 0.299 | 0.505±0.009 | 0.417±0.028 | 0.442±0.015 | 0.129 | 0.436±0.013 |
| Lunch input frequency (n/person-day) | 0.612±0.024 | 0.605±0.015 | 0.663 | 0.607±0.013 | 0.566±0.035 | 0.536±0.018 | 0.134 | 0.543±0.016 | 0.442±0.021 | 0.461±0.01 | 0.123 | 0.457±0.009 | 0.365±0.026 | 0.403±0.014 | 0.014 | 0.394±0.012 |
| Dinner input frequency (n/person-day) | 0.558±0.025 | 0.546±0.015 | 0.406 | 0.55±0.013 | 0.488±0.035 | 0.463±0.017 | 0.208 | 0.468±0.016 | 0.366±0.02 | 0.381±0.009 | 0.162 | 0.378±0.009 | 0.298±0.025 | 0.332±0.013 | 0.017 | 0.325±0.012 |
| Breakfast calories (kcal/person-day) | 314.539±9.742 | 263.457±4.819 | <0.001 | 278.015±4.53 | 337.237±14.011 | 273.644±5.465 | <0.001 | 287.877±5.445 | 342.946±8.585 | 284.892±3.429 | <0.001 | 296.277±3.305 | 327.875±11.698 | 279.686±5.33 | <0.001 | 290.566±4.989 |
| Lunch calories (kcal/person-day) | 463.721±11.105 | 371.17±5.631 | <0.001 | 397.546±5.415 | 495.396±15.287 | 391.509±6.438 | <0.001 | 414.761±6.448 | 510.974±10.119 | 405.487±3.959 | <0.001 | 426.173±3.963 | 498.777±14.228 | 387.222±6.035 | <0.001 | 412.407±6.081 |
| Dinner calories (kcal/person-day) | 555.072±13.632 | 429.976±6.819 | <0.001 | 465.627±6.672 | 582.211±19.969 | 444.954±7.833 | <0.001 | 475.674±8.093 | 594.568±12.815 | 446.516±4.699 | <0.001 | 475.549±4.88 | 566.945±17.686 | 425.51±7.123 | <0.001 | 457.441±7.357 |
| * exercise related variables |  |  |  |  |  |  |  |  |  |  |  |  |  |  |  |  |
| Exercise input frequency (n/person-day) | 0.353±0.021 | 0.349±0.012 | 0.718 | 0.35±0.011 | 0.315±0.026 | 0.307±0.014 | 0.599 | 0.309±0.012 | 0.254±0.015 | 0.261±0.007 | 0.404 | 0.259±0.007 | 0.241±0.019 | 0.253±0.011 | 0.288 | 0.25±0.009 |
| Exercise calories consumption (kcal/person-day) | 414.718±16.768 | 291.359±7.582 | <0.001 | 326.515±7.597 | 417.174±26.572 | 292.208±9.572 | <0.001 | 320.178±9.88 | 413.702±16.781 | 276.393±5.305 | <0.001 | 303.319±5.638 | 409.213±22.22 | 275.075±8.164 | <0.001 | 305.359±8.49 |
| 3) Change of weight |  |  |  |  |  |  |  |  |  |  |  |  |  |  |  |  |
| Final BMI | 26.849±0.347 | 26.022±0.278 | <0.001 | 26.257±0.223 | 27.319±0.501 | 26.301±0.343 | 0.001 | 26.529±0.29 | 29.257±0.371 | 26.939±0.23 | <0.001 | 27.394±0.201 | 29.695±0.502 | 27.24±0.347 | <0.001 | 27.794±0.295 |
| Underweight (< 18.5) | 0.008 | 0.027 | <0.001 | 0.022 | 0.003 | 0.026 | <0.001 | 0.021 | 0.001 | 0.022 | <0.001 | 0.018 | 0 | 0.013 | - | 0.01 |
| Normal (18.5~25) | 0.359 | 0.483 | <0.001 | 0.448 | 0.318 | 0.479 | <0.001 | 0.443 | 0.204 | 0.471 | <0.001 | 0.418 | 0.149 | 0.439 | <0.001 | 0.373 |
| Overweight (25~30) | 0.436 | 0.287 | <0.001 | 0.329 | 0.456 | 0.28 | 0.287 | 0.32 | 0.465 | 0.253 | 0.003 | 0.295 | 0.452 | 0.287 | 0.125 | 0.324 |
| Obesity class I (30~35) | 0.143 | 0.13 | 0.09 | 0.134 | 0.153 | 0.125 | 0.001 | 0.131 | 0.199 | 0.136 | <0.001 | 0.148 | 0.252 | 0.146 | 1 | 0.17 |
| Obesity class II (35~40) | 0.044 | 0.052 | 0.005 | 0.05 | 0.043 | 0.059 | <0.001 | 0.055 | 0.087 | 0.067 | <0.001 | 0.071 | 0.095 | 0.07 | 0.062 | 0.075 |
| Obesity class III (>40) | 0.01 | 0.02 | 0.001 | 0.017 | 0.028 | 0.031 | 0.007 | 0.03 | 0.045 | 0.051 | <0.001 | 0.049 | 0.051 | 0.046 | 0.01 | 0.047 |

Data are reported as the mean ± SD, unless otherwise stated.

** To be classified as the success group, users had to achieve weight loss of at least 10% of their baseline weight during the first 26 weeks (initial phase), and maintain the loss until the last observation, and the mean weight of the long-term phase was not to exceed the lowest weight in the initial phase. To be classified as the partial success group, users had to lose between 5% and 10% of their baseline weight during the initial phase and maintain the loss in the long-term phase. Users were classified as being in the stationary group if their maximum weight loss during the initial phase is less than 5% of their baseline weight and they maintain this level of weight loss until the end of the study period. To be classified as the yo-yo group, users had to lose at least 5% of their baseline weight but regain the weight. Weight regain was defined as a user whose weight at the last observation is greater than 97% of the baseline weight after having lost more than 5% of the baseline weight in the initial phase.

**Table 2.** Number of participants according to country

| country | Male | Female |
| --- | --- | --- |
| United States of America | 2672 | 8515 |
| Germany | 1659 | 7810 |
| Korea Republic | 738 | 4098 |
| Great Britain | 809 | 2002 |
| Japan | 397 | 1381 |
| Canada | 153 | 524 |
| Australia | 111 | 425 |
| Austria | 77 | 371 |
| Swiss | 64 | 259 |
| Netherlands | 53 | 258 |
| Spain | 88 | 221 |
| France | 56 | 196 |
| Itary | 77 | 161 |
| Russia | 45 | 143 |
| New Zealand | 37 | 122 |
| Republic of South Africa | 44 | 105 |
| Sweden | 39 | 93 |
| Ireland | 18 | 89 |
| India | 72 | 80 |
| Israel | 37 | 77 |
| Poland | 53 | 71 |
| Finland | 17 | 68 |
| Norway | 15 | 65 |
| Greece | 32 | 61 |
| Philippines | 29 | 60 |
| Brazil | 49 | 59 |
| Turkey | 23 | 56 |
| Denmark | 19 | 49 |
| Hungary | 19 | 48 |
| Slovenia | 16 | 45 |
| Taiwan | 11 | 43 |
| Belgium | 8 | 43 |
| Croatia | 11 | 41 |
| Portugal | 23 | 40 |
| Hong Kong | 12 | 34 |
| Romania | 10 | 32 |
| Latvia | 2 | 27 |
| Lithuania | 5 | 27 |
| United Arab Emirates | 3 | 23 |
| Sweden | 8 | 22 |
| Bulgaria | 5 | 22 |
| Mexico | 10 | 22 |
| Czech | 16 | 20 |
| Estonia | 6 | 19 |
| Ukranie | 5 | 13 |
| Thai | 13 | 12 |
| Slovakia | 8 | 11 |
| Argentina | 8 | 10 |
| Liechtenstein | 0 | 10 |
| China | 8 | 9 |
| Indonesia | 8 | 9 |
| Iceland | 3 | 6 |
| Malaysia | 6 | 6 |
| Serbia | 5 | 4 |
| Venezuela | 2 | 3 |
| Chile | 0 | 3 |
| Colombia | 0 | 3 |
| Vietnam | 1 | 3 |
| Luxembourg | 0 | 3 |
| Dominican Republic | 0 | 3 |
| Egypt | 1 | 3 |
| EN | 0 | 2 |
| Puerto Rico | 0 | 2 |
| Uruguay | 0 | 2 |
| Peru | 0 | 2 |
| Macedonia | 1 | 2 |
| Belarus | 0 | 2 |
| Iran | 1 | 1 |
| Guatemala | 0 | 1 |
| Saudi Arabia | 1 | 1 |
| Molta | 0 | 1 |
| Jordan | 0 | 1 |
| Bosnia and Herzegovina | 1 | 1 |
| FA | 0 | 1 |
| Kuwait | 1 | 0 |
| Serbia | 1 | 0 |
| Pakistan | 1 | 0 |
| Panama | 1 | 0 |
| Honduras | 1 | 0 |
| Bolivia | 1 | 0 |

**Table 3.** Linear weight reduction in the first 26 weeks of follow-up in success, partial success and stationary subgroups

|  | **Male** | | | **Female** | | |
| --- | --- | --- | --- | --- | --- | --- |
|  | ***β*** | **Standard Error** | **p-value** | ***β*** | **Standard Error** | **p-value** |
| success | -0.655 | 0.020 | <0.001 | -0.515 | 0.013 | <0.001 |
| partial success | -0.256 | 0.028 | <0.001 | -0.201 | 0.015 | <0.001 |
| stationary | -0.032 | 0.018 | 0.082 | 0.004 | 0.009 | 0.679 |

**Table 4.** Linear weight reduction in the first 8 weeks of follow-up in partial success and yoyo subgroups

|  | **Male** | | | **Female** | | |
| --- | --- | --- | --- | --- | --- | --- |
|  | ***β*** | **Standard Error** | **p-value** | ***β*** | **Standard Error** | **p-value** |
| partial success | -0.437 | 0.169 | 0.010 | -0.295 | 0.089 | 0.001 |
| yo-yo | -0.464 | 0.132 | <0.001 | -0.365 | 0.078 | <0.001 |

A.


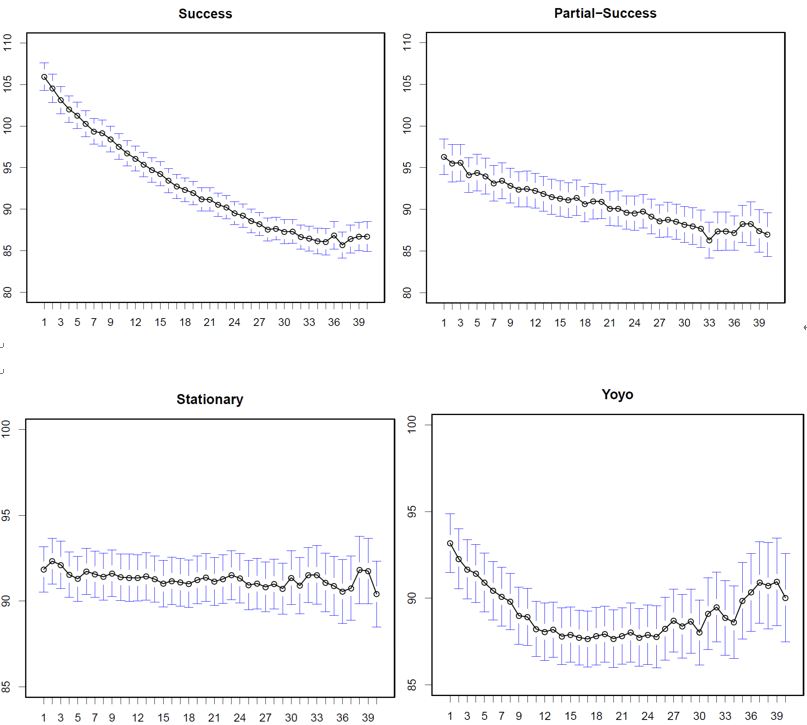


B.


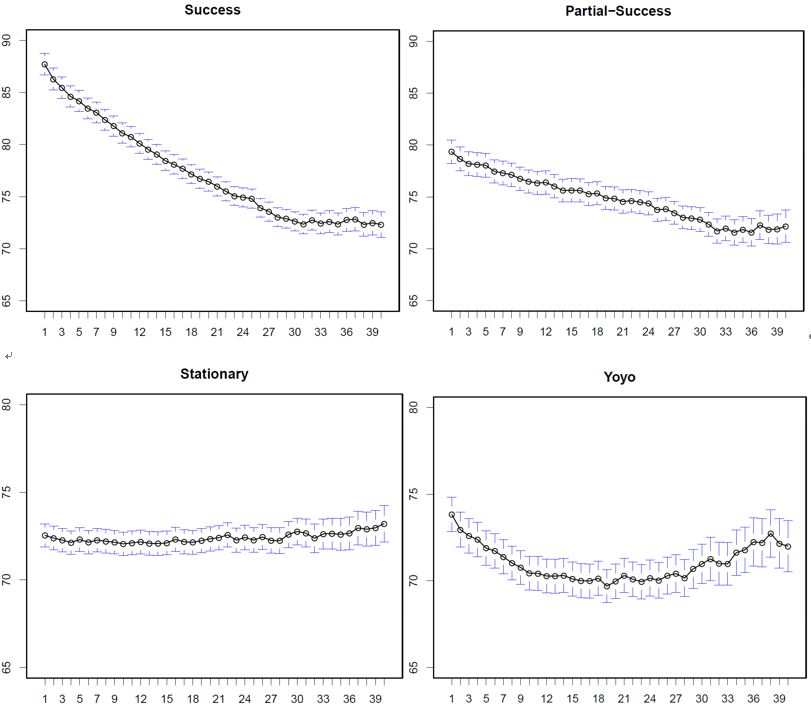


**Figure 1.** Weight change patterns of the four subgroups: A. male; B. female. Data are presented as the mean ± SD.

**Table 5. Risk factors contributing to being a yo-yo against stationary subgroup**

|  | Univariate Logistic Regression |  | Multivariate Logistic Regression |  |
| --- | --- | --- | --- | --- |
|  | OR (95% CI) | Wald Test  p-value | OR (95% CI) | Wald Test  p-value |
| Gender (male) | 1.20 (1.04, 1.37) | 0.011 | 1.43 (1.22, 1.68) | <0.001 |
| Age | 0.98 (0.97, 0.99) | <0.001 | 0.98 (0.97, 0.99) | <0.001 |
| Follow-up Days | 1.00 (1.00, 1.01) | <0.001 | 1.00 (1.00, 1.00) | <0.001 |
| Baseline BMI | 1.01 (1.00, 1.02) | 0.005 | 1.02 (1.01, 1.03) | 0.001 |
| Weight input frequency (n/person-day) | 0.19 (0.13, 0.28) | <0.001 | 0.59 (0.39, 0.90) | 0.013 |
| Breakfast input frequency (n/person-day) | 0.44 (0.36, 0.54) | <0.001 | 0.76 (0.42, 1.36) | 0.352 |
| Lunch input frequency (n/person-day) | 0.45 (0.37, 0.56) | <0.001 | 0.84 (0.36, 2.00) | 0.690 |
| Dinner input frequency (n/person-day) | 0.47 (0.38, 0.59) | <0.001 | 1.88 (0.94, 3.74) | 0.072 |
| Breakfast calories (kcal/person-day) | 1.00 (1.00, 1.00) | 0.064 | 1.00 (1.00, 1.00) | 0.900 |
| Lunch calories (kcal/person-day) | 1.00 (1.00, 1.00) | <0.001 | 1.00 (1.00, 1.00) | 0.021 |
| Dinner calories (kcal/person-day) | 01.00 (1.00, 1.00) | <0.001 | 1.00 (1.00, 1.00) | 0.022 |
| Exercise input frequency (n/person-day) | 0.81 (0.61, 1.06) | 0.119 | 1.06 (0.78, 1.45) | 0.707 |
| Exercise calories expenditure (kcal/person-day) | 1.00 (1.00, 1.00) | 0.698 | - | - |

Abbreviations: BMI, body mass index; OR, odds ratio; CI, confidence interval.
